# First-line osimertinib in elderly patients with epidermal growth factor receptor-mutated advanced non-small cell lung cancer: a retrospective multicenter study (HOT2002)

**DOI:** 10.1038/s41598-021-02561-z

**Published:** 2021-11-30

**Authors:** Gaku Yamamoto, Hajime Asahina, Osamu Honjo, Toshiyuki Sumi, Atsushi Nakamura, Kenichiro Ito, Hajime Kikuchi, Fumihiro Hommura, Ryoichi Honda, Keiki Yokoo, Yuka Fujita, Satoshi Oizumi, Ryo Morita, Yasuyuki Ikezawa, Hisashi Tanaka, Nozomu Kimura, Takaaki Sasaki, Noriaki Sukoh, Taichi Takashina, Toshiyuki Harada, Hirotoshi Dosaka-Akita, Hiroshi Isobe, the Hokkaido Lung Cancer Clinical Study Group Trial, Gaku Yamamoto, Hajime Asahina, Osamu Honjo, Toshiyuki Sumi, Atsushi Nakamura, Kenichiro Ito, Hiroshi Isobe, Hajime Kikuchi, Fumihiro Hommura, Ryoichi Honda, Keiki Yokoo, Yuka Fujita, Satoshi Oizumi, Ryo Morita, Yasuyuki Ikezawa, Hisashi Tanaka, Nozomu Kimura, Takaaki Sasaki, Noriaki Sukoh, Taichi Takashina, Toshiyuki Harada, Hirotoshi Akita

**Affiliations:** 1 Department of Respiratory Medicine, Hokkaido University Graduate School of Medicine, North 15, West 7, Kita-ku, Sapporo, 060-8638 Japan; 2 Department of Respiratory Medicine, Sapporo Minami Sanjo Hospital, Sapporo, Japan; 3 Department of Pulmonary Medicine, Hakodate Goryoukaku Hospital, Hakodate, Japan; 4 Department of Pulmonary Medicine, Sendai Kousei Hospital, Sendai, Japan; 5 Department of Respiratory Medicine, KKR Sapporo Medical Center, Sapporo, Japan; 6 Department of Respiratory Medicine, Obihiro-Kosei General Hospital, Obihiro, Japan; 7 Department of Respiratory Medicine, Sapporo City General Hospital, Sapporo, Japan; 8 Department of Respiratory Medicine, Kokuho Asahi Chuo Hospital, Asahi, Japan; 9 Department of Respiratory Medicine, Teine Keijinkai Hospital, Sapporo, Japan; 10 Department of Respiratory Medicine, National Hospital Organization Asahikawa Medical Center, Asahikawa, Japan; 11 Department of Respiratory Medicine, National Hospital Organization Hokkaido Cancer Center, Sapporo, Japan; 12 Department of Respiratory Medicine, Akita Kousei Medical Center, Akita, Japan; 13 Department of Respiratory Medicine, Oji General Hospital, Tomakomai, Japan; 14 Department of Respiratory Medicine, Hirosaki University Graduate School of Medicine, Hirosaki, Japan; 15 Department of Respiratory Medicine, Tohoku University Graduate School of Medicine, Sendai, Japan; 16 Respiratory Center, Asahikawa Medical University, Asahikawa, Japan; 17 Department of Respiratory Medicine, National Hospital Organization Hokkaido Medical Center, Sapporo, Japan; 18 Department of Respiratory Medicine, Iwamizawa Municipal General Hospital, Iwamizawa, Japan; 19 Center for Respiratory Diseases, JCHO Hokkaido Hospital, Sapporo, Japan; 20 Department of Medical Oncology, Hokkaido University Graduate School of Medicine, Sapporo, Japan

**Keywords:** Targeted therapies, Non-small-cell lung cancer, Oncogenes

## Abstract

Osimertinib is a standard of care therapy for previously untreated epidermal growth factor receptor mutation-positive non-small cell lung cancer. However, limited data exist regarding the efficacy and safety of osimertinib as a first-line therapy for elderly patients aged 75 years or older. To assess the potential clinical benefits of osimertinib in this population, this retrospective multi-institutional observational study included 132 patients with non-small cell lung cancer (age ≥ 75 years), who received osimertinib as first-line treatment. The proportion of patients with 1-year progression-free survival was 65.8% (95% confidence interval 57.1–73.5). The median progression-free survival was 19.4 (95% confidence interval 15.9–23.9) months. The median overall survival was not reached (95% confidence interval 24.6–not reached). The frequency of pneumonitis was 17.4%, with a grade 3 or higher rate of 9.1%. More than two-thirds of treatment discontinuations due to pneumonitis occurred within 3 months of starting osimertinib, and the prognosis of patients with pneumonitis was unsatisfactory. Osimertinib is one of the effective first-line therapeutic options for patients aged 75 years or older; however, special caution should be exercised due to the potential development of pneumonitis.

## Introduction

Lung cancer is the leading cause of cancer-related deaths worldwide^1^. Non-small cell lung cancer (NSCLC) accounts for approximately 80% of lung cancers, most of which are advanced by the time of diagnosis^2,3^. In recent years, with an aging global population, the number of elderly patients with lung cancer is also increasing^4^. In fact, approximately 70% and 37% of patients newly diagnosed with lung cancer are over the age of 65 and 75 years, respectively. Meanwhile, patients aged 75 years or older comprise only 9% of clinical trial participants^5^. Generally, elderly patients with cancer tend to have more significant comorbidities and are more frail than non-elderly patients^6^. Therefore, it is crucial to develop a more optimum treatment strategy for this population.

Recent developments in NSCLC therapies that specifically target driver oncogenes, such as epidermal growth factor receptor tyrosine kinase inhibitor (EGFR-TKI)-sensitizing mutations, have changed the standard of care and prognosis for patients with advanced NSCLC^7–10^. EGFR-TKI-sensitizing mutations have been observed in approximately 50% of lung adenocarcinoma patients in Asian populations and 20% in Western populations^2^. Recently, a third-generation EGFR-TKI, osimertinib, has become a new standard of care for previously untreated EGFR mutation-positive NSCLCs, according to the FLAURA trial. The trial results showed more favorable progression-free survival (PFS) and overall survival (OS) with osimertinib than with first-generation EGFR-TKIs^9,11^. Although a subset analysis of patients aged 65 years or older was performed in the FLAURA trial, no previous study has been designed specifically for patients aged 75 years or older. A previous phase II study of patients with T790M mutation resistance to first-line EGFR-TKIs reported comparable efficacy and safety of osimertinib between non-elderly patients and those over 75 years of age^12^. However, the results of the study, after the acquisition of T790M mutation resistance, could not be directly applied to primary treatment as patients who were previously tolerant to EGFR-TKIs were included in the study. In addition, patients with poor performance status (PS) were excluded from these trials, resulting in a paucity of data on the safety and efficacy of osimertinib as the first-line treatment in this elderly population^9,12^.

Therefore, the aim of the current study was to describe the clinical benefits of first-line osimertinib in elderly patients with NSCLC. To this end, we retrospectively evaluated the clinical outcomes in patients aged 75 years or older with advanced NSCLC harboring EGFR-TKI-sensitizing mutations who received first-line osimertinib.

## Results

### Patient characteristics

The case report form for 147 patients was collected, of whom 2 patients with stage I or II, 1 patient aged 74 years old at the start of osimertinib treatment, and 12 patients starting osimertinib after January 2020, were excluded from the analysis. Ultimately, 132 patients (94 females, 38 males; median age, 80 [range 75–90] years) were included in the analysis. Patient characteristics are summarized in Table 1. Of the 132 patients, 92 reported having never smoked. A total of 93 patients (70.5%) had stage IV NSCLC, and 34 (25.8%) had a postoperative or post-radiotherapy recurrence. A total of 98 patients (74.2%) had a CCI of 0 or 1. Detailed data on the comorbidities of the study participants at baseline are shown in Table 2. Moreover, 113 (85.6%) had a PS of 0–1, 15 (11.4%) had a PS of 2, 3 (2.3%) had a PS of 3, and 1 (0.8%) had a PS of 4. Patients with PS ≥ 2 had significantly more advanced stage and liver metastasis than those with PS 0–1, though there was no apparent difference in CCI (Supplementary Table S1). Therefore, the cause of worsening of PS in our study population could be attributed to the primary disease rather than to comorbidity. The histological type was adenocarcinoma in all patients. Regarding the type of EGFR mutation, 46 (34.9%) and 80 (60.6%) patients exhibited an exon 19 deletion and exon 21 L858R mutation (including patients with concurrent T790M or other uncommon mutations), respectively. One patient had both an exon 19 deletion and L858R mutation. Five patients had uncommon mutations, including G719X, L861Q, and exon 20 insertions.

**Table 1:** Baseline characteristics of patients aged 75 years or older with NSCLC who were treated with osimertinib as first-line therapy (n = 132).

Characteristic	n
Age, median (range), years	80 (75–90)
**Age group, n (%)**	
75–79 years	63 (47.7)
80–84 years	44 (33.3)
≥ 85 years	25 (18.9)
**Sex, n (%)**	
Female	94 (71.2)
Male	38 (28.8)
**Pathology, n (%)**	
Adenocarcinoma	132 (100)
**Stage, n (%)**	
IIIA	2 (1.5)
IIIB	3 (2.3)
IVA	37 (28.0)
IVB	56 (42.4)
Recurrence	34 (25.8)
**ECOG performance status, n (%)**	
0	30 (22.7)
1	83 (62.9)
2	15 (11.4)
3	3 (2.3)
4	1 (0.8)
**CNS metastasis, n (%)**	
Present	27 (20.5)
Absent	105 (79.5)
**Liver metastasis, n (%)**	
Present	10 (7.6)
Absent	122 (92.4)
**Smoking history, n (%)**	
Yes	40 (30.3)
No	92 (69.7)
**EGFR mutation, n (%)**	
Exon 19 deletion	45 (34.1)
Exon 19 deletion + T790M	1 (0.8)
L858R	75 (56.8)
L858R + T790M	4 (3.0)
L858R + S768I	1 (0.8)
Exon 19 deletion + L858R	1 (0.8)
G719X	3 (2.3)
L861Q	1 (0.8)
Exon 20 insertion	1 (0.8)
**CCI, n (%)**	
CCI < 2	98 (74.2)
CCI ≥ 2	34 (25.8)
**BSA (Du Bois Method), n (%)**	
BSA < 1.5 m^2^	88 (66.7)
BSA ≥ 1.5 m^2^	42 (31.8)
NA	2 (1.5)

*BSA* body surface area, *CCI* Charlson comorbidity index, *CNS* central nervous system, *ECOG* Eastern Cooperative Oncology Group, *EGFR* epidermal growth factor receptor, *NA* not available.

**Table 2:** The comorbidities at baseline based on the Charlson comorbidity index (n = 132).

Comorbidity	n (%)
Diabetes^a^	17 (12.9)
Solid cancers other than lung cancer^b^	17 (12.9)
Dementia	11 (8.3)
Cerebrovascular disorder	9 (6.8)
Congestive heart failure^c^	6 (4.5)
Peptic ulcer disease	5 (3.8)
Chronic lung disease^d^	4 (3.0)
Moderate to severe renal disease^e^	4 (3.0)
Liver disease	3 (2.3)
Peripheral vascular disease	2 (1.5)
Myocardial infarction	1 (0.8)
Hematologic disease (Lymphoma, Leukemia, AIDS)	1 (0.8)
Connective tissue disease	0

^a^Excluding patients treated without medication.

^b^Diagnosed within the past 5 years. Solid cancers that do not require treatment at the time of enrollment, or where lung cancer is believed to determine the prognosis.

^c^Exertional or paroxysmal nocturnal dyspnea and has responded to medications.

^d^Leading to dyspnea even with mild exertion.

^e^Severe = on dialysis, status post kidney transplant, uremia, moderate = creatinine > 3 md/dL.

### Treatment efficacy and survival

**Figure 1:**
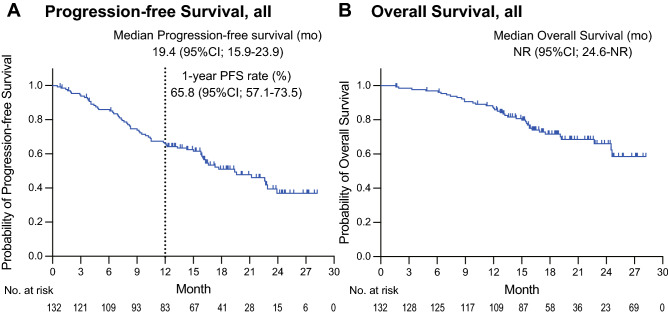
Kaplan–Meier curves of (**A**) progression-free survival (PFS) and (**B**) overall survival from osimertinib initiation in all patients. *CI* confidence interval, *NR* not reached.

At the median follow-up time of 20.5 (range 12.3–28.3) months, the primary endpoint (i.e., 1-year PFS) was 65.8% (95% CI 57.1–73.5; Fig. 1A). The median PFS and TTF were 19.4 (95% CI 15.9–23.9) months and 17.7 (95% CI 14.3–22.9) months, respectively (Fig. 1A and Supplementary Fig. S1). The median OS was not reached (NR; 95% CI 24.6–NR; Fig. 1B). Among the 113 (85.6%) cases with measurable lesions, the ORR and DCR were 75.2% (95% CI 66.5–82.3) and 92.9% (95% CI 86.6–96.3%), respectively. There were 3 patients with complete response, 82 with partial response, 20 with stable disease, and 2 with progressive disease. Table 3 presents the PFS from the initiation of osimertinib in patients according to different subgroups. No significant difference was observed in efficacy between patients aged ≥ 80 and < 80 years, or between CCI 0–1 and ≥ 2, whereas PS 2 or worse was identified as a poor prognostic factor. Patients with exon 19 deletions tended to have a better PFS than those with the L858R mutation (Supplementary Fig. S2; PFS, EGFR exon 19 del vs L858R). The Kaplan–Meier curves of PFS according to EGFR common and uncommon mutation are shown in Supplementary Fig. S3. Univariate analyses showed that a PS of 2 or worse was significantly associated with shorter PFS (HR 1.96; 95% CI 1.01–3.78; *p* = 0.044). No other significant factors were identified in the univariate or multivariate analyses (Supplementary Table S2).

**Table 3:** Progression-free survival (PFS) according to different subgroups.

Subgroup	n	Median (95% CI) PFS, months	*p* value (log-rank)
**Age, years**			
75–79	63	22.7 (15–NR)	0.66
≥ 80	69	19.4 (10.4–22.9)	
**Sex**			
Male	38	19.6 (13.3–NR)	0.89
Female	94	19.4 (15.8–NR)	
**Smoking history**			
Yes	40	16.1 (9.5–NR)	0.61
No	92	21.2 (15.9–NR)	
**Clinical stage**			
III or IV	98	16.3 (13.3–22.6)	0.139
Recurrence^a^	34	22.9 (16.6–NR)	
**Performance status**			
0–1	113	21.2 (15.8–NR)	0.040
≥ 2	19	16.1 (4.1–17.7)	
**Brain metastasis**			
Present	27	17.3 (11.8–21.2)	0.46
Absent	105	22.6 (15.8–NR)	
**EGFR mutation**^b^			
Exon 19 deletion	44	22.6 (15.8–NR)	0.69
L858R	80	16.6 (12.2–NR)	
**CCI**			
0–1	98	19.6 (15.9–23.9)	0.87
≥ 2	34	17.3 (10.5–NR)	
**BSA**^c^			
< 1.5	88	19.4 (13.3–23.9)	0.50
≥ 1.5	42	19.6 (15–NR)	

*BSA* body surface area, *CCI* Charlson comorbidity index, *CI* confidence interval, *EGFR* epidermal growth factor, receptor, *NR* not reached.

^a^Recurrence after complete resection or curative-intent (chemo) radiotherapy.

^b^Patients could have more than one type of mutation. A patient who has compound mutation of exon 19 deletion and L858R was excluded from this subgroup analysis.

^c^Patients whose data on height or weight were not available were excluded from this subgroup analysis.

**Figure 2:**
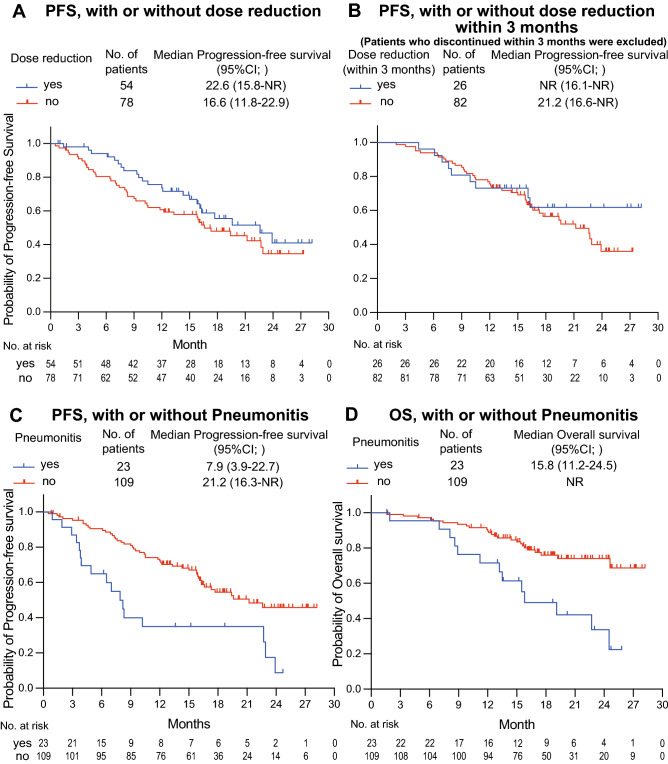
Kaplan–Meier curves of PFS in patients (**A**) who had dose reduction and those who remained on daily 80 mg osimertinib; (**B**) those who had dose reductions within the first 3 months and those who remained on daily 80 mg osimertinib for the first 3 months. All patients for whom treatment was discontinued within the first 3 months were excluded. (**C**) Kaplan–Meier curves of PFS in patients with or without pneumonitis. (**D**) Kaplan–Meier curves of OS in patients with or without pneumonitis. *CI* confidence interval, *NR* not reached, *PFS* progression-free survival.

**Figure 3:**
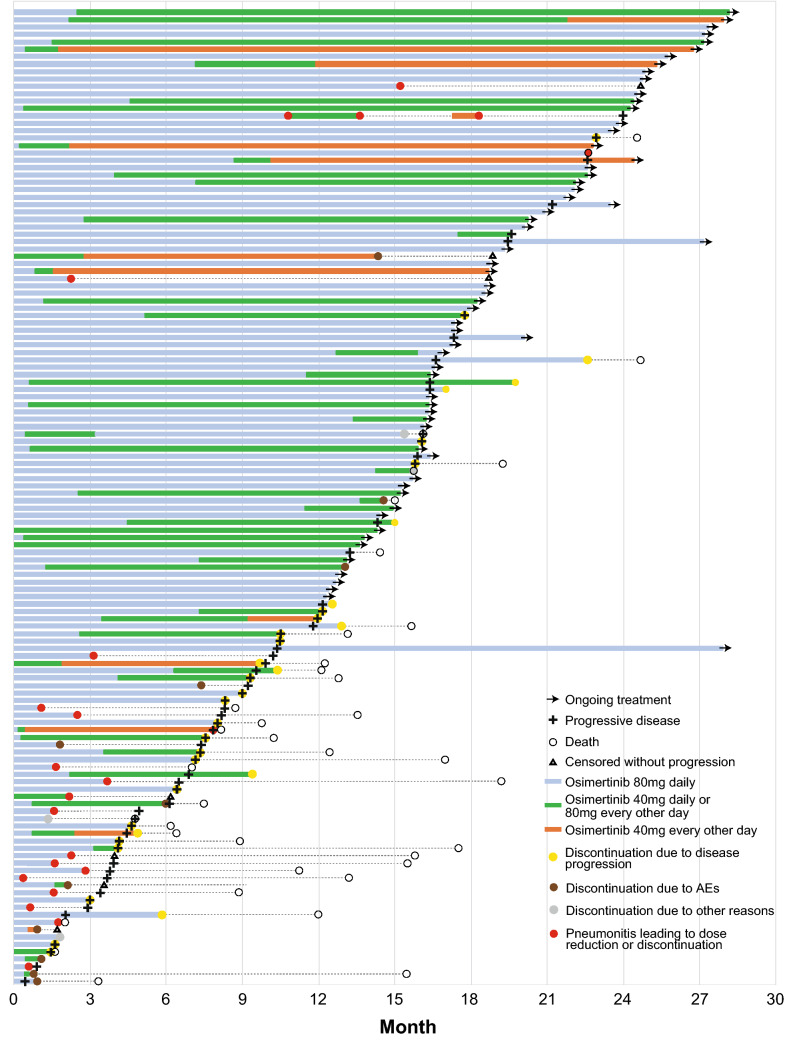
Swimmer plot for progression-free survival, overall survival, treatment duration, osimertinib dose, and reasons leading to treatment discontinuation in all patients (n = 132). *AEs* adverse events.

Although no statistically significant difference was noted, PFS in patients treated with a reduced dose was numerically better than in those without dose reduction (Fig. 2A). To examine the effect of dose reduction on PFS, we compared patients who experienced dose reduction within 3 months of initiating osimertinib (early dose reduction group) and others (non-early dose reduction group). To reduce potential selection bias, data from patients who discontinued treatment within 3 months were excluded. The Kaplan–Meier curves of the early and non-early dose reduction groups were nearly identical (Fig. 2B). Patients in the early dose reduction group had PFS and treatment responses comparable to those in the non-early dose reduction group (Fig. 3 and Supplementary Fig. S4; waterfall plot). An analysis that included patients who discontinued osimertinib within 3 months showed similar results (Supplementary Fig. S5; PFS with or without dose reduction within 3 months). Meanwhile, pneumonitis during osimertinib treatment had a poor prognostic impact. As indicated in Table 4, 23 patients experienced pneumonitis (any grade) with an associated median PFS of 7.9 months (95% CI 3.9–22.7) compared to 21.2 months (95% CI 16.3–NR) in patients without pneumonitis (Fig. 2C). The median OS was 15.8 months (95% CI 11.2–24.5) in patients with pneumonitis and NR in patients without pneumonitis (Fig. 2D). Additionally, the median TTF in the pneumonitis group was 2.3 months (95% CI 1.6–3.7; Supplementary Fig. S6), and 70.0% (14 of 20) of treatment discontinuations due to pneumonitis occurred within 3 months of initiating osimertinib (Fig. 3).

**Table 4:** Adverse events during osimertinib treatment (n = 132).

Adverse event^a^	Grade 1	Grade 2	Grade 3	Grade 4	Grade 5	Any grade	Grade ≥ 3
	n (%)						
Rash or acne^b^	35 (26.5)	17 (12.9)	0	0	0	52 (39.4)	0
Paronychia	39 (29.5)	17 (12.9)	2 (1.5)	0	0	58 (43.9)	2 (1.5)
Dry skin	45 (34.1)	6 (4.5)	0	0	0	51 (38.6)	0
Pruritus	21 (15.9)	1 (0.8)	0	0	0	22 (16.7)	0
Oral mucositis	18 (13.6)	5 (3.8)	1 (0.8)	0	0	24 (18.2)	1 (0.8)
Diarrhea	33 (25.0)	7 (5.3)	0	0	0	40 (30.3)	0
Constipation	7 (5.3)	0	0	0	0	7 (5.3)	0
Nausea	6 (4.5)	7 (5.3)	5 (3.8)	0	0	18 (13.6)	5 (3.8)
Vomiting	4 (3.0)	2 (1.5)	1 (0.8)	0	0	7 (5.3)	1 (0.8)
Fatigue	23 (17.4)	9 (6.8)	1 (0.8)	0	0	33 (25.0)	1 (0.8)
Anorexia	9 (6.8)	10 (7.6)	11 (8.3)	0	0	30 (22.7)	11 (8.3)
Dyspnea	4 (3.0)	4 (3.0)	3 (2.3)	0	0	11 (8.3)	3 (2.3)
Fever	5 (3.8)	0	1 (0.8)	0	0	6 (4.5)	1 (0.8)
Lung infection	1 (0.8)	1 (0.8)	2 (1.5)	0	0	4 (3.0)	2 (1.5)
Pneumonitis	5 (3.8)	6 (4.5)	8 (6.1)	1 (0.8)	3 (2.3)	23(17.4)	12 (9.1)
Prolonged QTc interval	4 (3.0)	0	0	0	0	4 (3.0)	0
Heart failure	0	3 (2.3)	2 (1.5)	0	1 (0.8)	6 (4.5)	3 (2.3)
Hypoalbuminemia	32 (24.2)	12 (9.1)	0	0	0	44 (33.3)	0
AST increased	26 (19.7)	5 (3.8)	2 (1.5)	0	0	33 (25.0)	2 (1.5)
ALT increased	27 (20.5)	5 (3.8)	1 (0.8)	0	0	33 (25.0)	1 (0.8)
Cre increased	18 (13.6)	10 (7.6)	1 (0.8)	0	0	29 (22.0)	1 (0.8)
CPK increased	11 (8.3)	1 (0.8)	2 (1.5)	1 (0.8)	0	15 (11.4)	3 (2.3)
Anemia	33 (25.0)	14 (10.6)	4 (3.0)	0	0	51 (38.6)	4 (3.0)
WBC decreased	11 (8.3)	5 (3.8)	1 (0.8)	0	0	17 (12.9)	1 (0.8)
Neutrophil count decreased	7 (5.3)	5 (3.8)	1 (0.8)	0	0	13 (9.8)	1 (0.8)
Platelet count decreased	25 (18.9)	3 (2.3)	0	0	0	28 (21.2)	0

*ALT* alanine aminotransferase, *AST* aspartate aminotransferase, *CPK* creatine phosphokinase, *Cre* creatinine, *WBC* white blood cell.

^a^Adverse events were graded using the National Cancer Institute Common Terminology Criteria for Adverse Events, version 5.0.

^b^This category represents a grouped term for the event.

### Adverse events

All recorded AEs that occurred during first-line osimertinib therapy are summarized in Table 4. AEs of grade 3 or higher were reported in 41.7% of patients. The most common AE was paronychia (43.9%; grade ≥ 3 in 1.5%), followed by rash or acne (39.4%; grade ≥ 3 in 0%), dry skin (38.6%; grade ≥ 3 in 0%), and anemia (38.6%; grade ≥ 3 in 3.0%). The frequency of pneumonitis was 17.4%, with a grade 3 or higher rate of 9.1%. The mortality rate of pneumonitis during osimertinib was 13.0% (3 of 23 cases). The median time to discontinuation of osimertinib due to pneumonitis was 2.2 (95% CI 1.4–3.1) months. There was no difference in baseline patient background between patients with or without pneumonitis (Supplementary Table S3).

Fifty-four patients (40.9%) required at least one dose reduction of osimertinib due to AEs (Supplementary Table S4). The most frequent causes of dose reduction were anorexia, rash, and diarrhea (17.0%, 11.4%, and 9.1%, respectively). Thirty-five patients (26.5%) discontinued osimertinib, with pneumonitis reported as the most common cause of discontinuation (Supplementary Table S5). There were four treatment-related deaths (three due to pneumonitis and one due to heart failure; Supplementary Table S6).

### Treatment after disease progression

Of the 65 patients with disease progression after starting osimertinib, 5 were continuing first-line osimertinib beyond progression at the data cut-off date. Thirty-three patients had received second-line treatment. Cytotoxic chemotherapy was performed in 22 patients; 9 patients received platinum doublet (with or without antiangiogenic agent), and 12 patients received monotherapy. Eight patients received re-administration of EGFR-TKIs. Two patients received an immune checkpoint inhibitor with or without combined chemotherapy. One patient received salvage surgery.

## Discussion

To the best of our knowledge, this is the first real-world study of elderly patients aged 75 years or older who received osimertinib as first-line treatment for advanced EGFR-mutant NSCLC. We observed a 1-year PFS of 65.8%, median PFS of 19.4 months, ORR of 75.2%, and DCR of 92.9%, all of which are comparable with those of the FLAURA trial in which osimertinib showed efficacy with 1-year PFS of approximately 70% and median PFS of 18.9 months. The safety profile of osimertinib is similar to that previously reported except for the occurrence of pneumonitis^9^. Although 40.9% of patients required dose reduction of osimertinib, there was no apparent difference in efficacy between patients with and without dose reduction. The frequency of pneumonitis was 17.4%, and the median OS of patients who had developed pneumonitis was poor (15.8 months). These data suggest that osimertinib as first-line therapy is one of the effective therapeutic options for this population; however, particular attention should be paid to the development of pneumonitis.

Elderly patients with NSCLC tend to have vulnerable clinical profiles, including lower organ function, polypharmacy, or comorbidities, resulting in their often being excluded from prospective clinical trials^4^. Meanwhile, previous prospective and retrospective studies evaluating the efficacy and safety of gefitinib, erlotinib, and afatinib as first-line treatments reported first- and second-generation EGFR-TKIs to be effective in the elderly patients with manageable toxicity (Supplementary Table S7)^13–24^. In fact, the NEJ003 study is the first to report the efficacy and safety of gefitinib in elderly patients aged 75 years or older with a median PFS of 12.3 months^13^. For osimertinib, a phase II clinical trial was conducted in elderly patients with EGFR T790M mutation-positive NSCLC with resistance to prior EGFR-TKI. The study demonstrated that the efficacy and safety were comparable to those in the non-elderly population^12^. A small retrospective study conducted at a single institution also showed similar results^25^. However, no previous data on the efficacy and safety of osimertinib as first-line treatment in elderly patients are available.

In this study, the rates of dose reduction and treatment discontinuation due to AEs (40.9% and 26.5%, respectively) in patients were higher than those reported in the FLAURA trial (4% and 13%, respectively). This reflects the fact that our cohort comprised real-world elderly patients, many of whom were not eligible for prospective clinical trials due to their vulnerable profiles^26,27^. Meanwhile, the patients who were treated with reduced doses yielded numerically better PFS than those who were not. Although this may include a bias that patients who were able to take the drug for a long period were more likely to experience AEs leading to dose reduction, the same tendency was observed when the analysis was limited to early dose reduction, i.e., within 3 months of initiating osimertinib. In the AURA study, which evaluated the safety and efficacy of osimertinib at doses of 20–240 mg once daily in patients with NSCLC harboring EGFR mutations and disease progression after previous treatment with EGFR-TKIs, the response rate was similar at doses of 20 mg, 40 mg, and 80 mg daily^28^. Previous studies of first- and second-generation EGFR-TKIs have shown that dose reduction allows for efficacy and tolerability in elderly or frail patients^21,23^. We, therefore, believe that appropriate dose reduction is necessary for the continuation of osimertinib, contributing to PFS prolongation.

The pattern of AEs reported in this study was relatively consistent with the known safety profile of osimertinib characterized in the FLAURA trial^9^. Osimertinib has a limited effect on wild-type EGFR and tends to have fewer side effects of skin rash and diarrhea than gefitinib, erlotinib, or afatinib^8,9^. In our study, the rates of grade ≥ 3 AEs, including diarrhea, paronychia, stomatitis, and rash, were reported in less than 5% of the patients. Meanwhile, the frequency of pneumonitis in the current study appeared higher than that previously reported^29^. The shorter PFS in patients with pneumonitis may be due to reduced dose intensity or due to the fact that frequent imaging for toxicity assessment can capture disease progression earlier than routine imaging. Generally, pneumonitis is a well-known adverse event associated with EGFR-TKIs, and the incidence of TKI-associated pneumonitis is higher in the Japanese population than in Western populations^30^. Although there were no obvious demographic differences between the patients with and without pneumonitis in this study, male sex, advanced age, smoking history, pre-existing pulmonary fibrosis, and poor PS have been suggested as potential risk factors for pneumonitis in TKI-treated patients^31^. In addition, osimertinib may be associated with a higher frequency of pneumonitis than first- and second-generation EGFR-TKIs^32^. Since the incidence of pneumonitis is an important poor prognostic factor, further studies are needed to clarify the risk factors for pneumonitis during osimertinib treatment.

The strengths of our study include the patient demographic characteristics, which reflect a fragile elderly population and detailed data on dose reduction and discontinuation, in real-world clinical practice. However, our study has several limitations. First, there is a lack of centrally independent verification of radiographic outcomes to confirm local investigator assessment. Second, due to the retrospective nature of the study, the timing of tumor assessment was not uniform among the study sites. This can lead to an over- or underestimation of PFS. However, it has been reported that PFS and TTF are highly correlated in a real-world evidence-based study^33^. Since PFS and TTF were relatively identical in our study, the validity of the results was confirmed. Third, we did not collect detailed information on the causal relationship between osimertinib and AEs. In this study, the number of patients who experienced AEs leading to dose reduction and discontinuation was higher than previously reported; however, the causal relationship with osimertinib is partially unclear. In addition, it is possible that we overestimated the frequency of pneumonitis because there are several conditions that can mimic pneumonitis, transient asymptomatic pulmonary opacities (TAPO) being one example^34^. In this study, we could not differentiate between these mimicking conditions and osimertinib-induced pneumonitis due to the lack of CT images when pneumonitis occurred. Finally, in retrospective observational studies, the heterogeneity in baseline characteristics of the patients other than age may have affected the results^35^. A prospective phase II study is ongoing to explore the role of first-line osimertinib in elderly patients (jRCTs071180007).

## Conclusion

Our study showed that osimertinib as first-line therapy is effective in patients aged 75 years or older, and is tolerable with appropriate dose reduction, with the exception of pneumonitis. However, the high frequency of pneumonitis, which was associated with poor prognosis, is a serious concern. Further large prospective studies are needed to assess the association of drug-related toxicities, especially pneumonitis, and baseline clinical characteristics.

## Materials and methods

### Study design and treatment

The Hokkaido Lung Cancer Clinical Study Group Trial 2002 (HOT2002) was a retrospective, observational, multicenter study conducted at 19 institutions in Japan. The objective of this study was to describe the clinical characteristics and outcomes of elderly patients with advanced NSCLC harboring EGFR mutations who received first-line osimertinib in a real-world setting. The study was conducted in accordance with the Declaration of Helsinki and approved by the institutional review boards (IRBs) of Hokkaido University Hospital, Sapporo Minami Sanjo Hospital, Hakodate Goryoukaku Hospital, Sendai Kousei Hospital, KKR Sapporo Medical Center, Obihiro-Kosei General Hospital, Sapporo City General Hospital, Kokuho Asahi Chuo Hospital, Teine Keijinkai Hospital, National Hospital Organization Asahikawa Medical Center, National Hospital Organization Hokkaido Cancer Center, Akita Kousei Medical Center, Oji General Hospital, Hirosaki University Graduate School of Medicine, Tohoku University Graduate School of Medicine, Asahikawa Medical University, National Hospital Organization Hokkaido Medical Center, Iwamizawa Municipal General Hospital, and JCHO Hokkaido Hospital. The requirement for informed consent was waived by all the approving IRBs due to the retrospective nature of the study. This study was registered at UMIN-CTR (UMIN000044101).

We evaluated consecutive elderly patients aged 75 years or older with advanced NSCLC harboring EGFR-TKI-sensitizing mutations who had started first-line osimertinib treatment between August 2018 and December 2019. Eligibility criteria were histologically- or cytologically-confirmed NSCLC, tumors with EGFR-TKI-sensitizing mutation, and aged 75 years or older when starting the first-line osimertinib. Patients who had received other EGFR-TKIs before receiving osimertinib were excluded.

### Data collection

Patient demographics and clinical characteristics, including age, sex, smoking status, tumor histology, cancer stage, number and sites of metastases, type of EGFR mutations, PS, height, weight, and Charlson comorbidity index (CCI), were obtained retrospectively from the patient files. Treatment exposure to osimertinib was also collected, including duration, doses, dose reduction, and discontinuation. The data cut-off date was December 31, 2020.

### Study endpoints and statistical analysis

The primary endpoint was the proportion of patients who had progression-free survival at 1 year (1-year PFS). The secondary endpoints were safety profile, objective response rate (ORR), disease control rate (DCR), PFS, time to treatment failure (TTF), and OS. Reported adverse events (AEs) during osimertinib treatment were graded according to the Common Terminology Criteria for AEs (version 5.0). Radiographic tumor responses were defined according to the Response Evaluation Criteria in Solid Tumors version 1.1.

PFS, OS, and TTF were estimated using the Kaplan–Meier method, and the log-rank test was used for inter-group comparisons. Hazard ratios (HRs) and 95% confidence intervals (CIs) were estimated using the Cox proportional hazards regression model. Patients who had uncommon mutations alone and who had a compound mutation with exon 19 deletion and L858R were excluded from the Cox proportional hazards analysis. Without considering the results of the univariate analysis, the factors considered important from the results of the previous report and medical point of view were selected for inclusion in the multivariate analysis. Categorical data were compared using Pearson’s chi-square test. Continuous data were compared using Wilcoxon rank-sum test. All *p*-values were two-sided, and the threshold for statistical significance was set at *p* < 0.05. All statistical analyses were performed using JMP software (version 15.2.0; SAS Institute, Cary, NC, USA) and GraphPad Prism version 9.1.0 (GraphPad Software, CA, USA).

## Supplementary Information

Supplementary Figures.

Supplementary Tables.

## Data Availability

The datasets used and/or analyzed during the current study are available from the corresponding author on reasonable request.

## References

[1] Fitzmaurice C (2018). Global, regional, and national cancer incidence, mortality, years of life lost, years lived with disability, and disability-adjusted life-years for 29 cancer groups, 1990 to 2016: A systematic analysis for the Global Burden of Disease Study. JAMA Oncol..

[2] Dearden S, Stevens J, Wu YL, Blowers D (2013). Mutation incidence and coincidence in non small-cell lung cancer: Meta-analyses by ethnicity and histology (mutMap). Ann. Oncol..

[3] Midha A, Dearden S, McCormack R (2015). EGFR mutation incidence in non-small-cell lung cancer of adenocarcinoma histology: A systematic review and global map by ethnicity (mutMapII). Am. J. Cancer Res..

[4] Pallis AG (2014). Management of elderly patients with NSCLC; updated expert's opinion paper: EORTC Elderly Task Force, Lung Cancer Group and International Society for Geriatric Oncology. Ann. Oncol..

[5] Marur S (2018). FDA analyses of survival in older adults with metastatic non-small cell lung cancer in controlled trials of PD-1/PD-L1 blocking antibodies. Semin. Oncol..

[6] Marosi C, Koller M (2016). Challenge of cancer in the elderly. ESMO Open..

[7] Maemondo M (2010). Gefitinib or chemotherapy for non-small-cell lung cancer with mutated EGFR. N. Engl. J. Med..

[8] Sequist LV (2013). Phase III study of afatinib or cisplatin plus pemetrexed in patients with metastatic lung adenocarcinoma with EGFR mutations. J. Clin. Oncol..

[9] Soria JC (2018). Osimertinib in untreated EGFR-mutated advanced non-small-cell lung cancer. N. Engl. J. Med..

[10] Rosell R (2012). Erlotinib versus standard chemotherapy as first-line treatment for European patients with advanced EGFR mutation-positive non-small-cell lung cancer (EURTAC): A multicentre, open-label, randomised phase 3 trial. Lancet Oncol..

[11] Ramalingam SS (2020). Overall survival with osimertinib in untreated, EGFR-mutated advanced NSCLC. N. Engl. J. Med..

[12] Nakao A (2019). Osimertinib in elderly patients with epidermal growth factor receptor t790m-positive non-small-cell lung cancer who progressed during prior treatment: A phase II trial. Oncologist..

[13] Maemondo M (2012). First-line gefitinib in patients aged 75 or older with advanced non-small cell lung cancer harboring epidermal growth factor receptor mutations: NEJ 003 study. J. Thorac. Oncol..

[14] Fujita S (2012). Customized chemotherapy based on epidermal growth factor receptor mutation status for elderly patients with advanced non-small-cell lung cancer: a phase II trial. BMC Cancer.

[15] Takahashi K (2014). First-line gefitinib therapy for elderly patients with non-small cell lung cancer harboring EGFR mutation: Central Japan Lung Study Group 0901. Cancer Chemother. Pharmacol..

[16] Uruga H (2010). Efficacy of gefitinib for elderly patients with advanced non-small cell lung cancer harboring epidermal growth factor receptor gene mutations: A retrospective analysis. Intern. Med..

[17] Tateishi K (2013). Clinical outcomes in elderly patients administered gefitinib as first-line treatment in epidermal growth factor receptor-mutated non-small-cell lung cancer: Retrospective analysis in a Nagano Lung Cancer Research Group study. Med. Oncol..

[18] Kuwako T (2015). First-line gefitinib treatment in elderly patients (aged >/=75 years) with non-small cell lung cancer harboring EGFR mutations. Cancer Chemother. Pharmacol..

[19] Morikawa N (2015). First-line gefitinib for elderly patients with advanced NSCLC harboring EGFR mutations. A combined analysis of North-East Japan Study Group studies. Expert Opin. Pharmacother..

[20] Inoue Y (2015). Phase II study of erlotinib in elderly patients with non-small cell lung cancer harboring epidermal growth factor receptor mutations. Cancer Chemother. Pharmacol..

[21] Miyamoto S (2020). Low-dose erlotinib treatment in elderly or frail patients with EGFR mutation-positive non-small cell lung cancer: A multicenter phase 2 trial. JAMA Oncol..

[22] Wu YL (2018). Afatinib as first-line treatment of older patients with EGFR mutation-positive non-small-cell lung cancer: Subgroup analyses of the LUX-Lung 3, LUX-Lung 6, and LUX-Lung 7 trials. Clin. Lung Cancer..

[23] Imai H (2018). A phase II study of afatinib treatment for elderly patients with previously untreated advanced non-small-cell lung cancer harboring EGFR mutations. Lung Cancer.

[24] Minegishi Y (2021). A phase II study of first-line afatinib for patients aged >/=75 years with EGFR mutation-positive advanced non-small cell lung cancer: North East Japan Study Group trial NEJ027. BMC Cancer.

[25] Kato Y (2019). Impact of clinical features on the efficacy of osimertinib therapy in patients with T790M-positive non-small cell lung cancer and acquired resistance to epidermal growth factor receptor tyrosine kinase inhibitors. J. Thorac. Dis..

[26] Ahmed Z, Kennedy K, Subramanian J (2021). The role for chemotherapy in 80 years and older patients with metastatic non-small cell lung cancer: A National cancer database analysis. Lung Cancer.

[27] Hsieh YY, Fang WT, Lo YW, Chen YH, Chien LN (2020). Comparing the effectiveness of different EGFR-TKIs in patients with EGFR mutant non-small-cell lung cancer: A retrospective cohort study in Taiwan. Int. J. Cancer..

[28] Janne PA (2015). AZD9291 in EGFR inhibitor-resistant non-small-cell lung cancer. N. Engl. J. Med..

[29] Ohe Y (2019). Osimertinib versus standard-of-care EGFR-TKI as first-line treatment for EGFRm advanced NSCLC: FLAURA Japanese subset. Jpn. J. Clin. Oncol..

[30] Gemma A (2020). Real-world evaluation of factors for interstitial lung disease incidence and radiologic characteristics in patients with EGFR T790M-positive NSCLC treated with osimertinib in Japan. J. Thorac. Oncol..

[31] Kudoh S (2008). Interstitial lung disease in Japanese patients with lung cancer: A cohort and nested case-control study. Am. J. Respir. Crit. Care Med..

[32] Oshima Y, Tanimoto T, Yuji K, Tojo A (2018). EGFR-TKI-associated interstitial pneumonitis in nivolumab-treated patients with non-small cell lung cancer. JAMA Oncol..

[33] Blumenthal GM (2019). Analysis of time-to-treatment discontinuation of targeted therapy, immunotherapy, and chemotherapy in clinical trials of patients with non-small-cell lung cancer. Ann. Oncol..

[34] Lee H (2018). Transient asymptomatic pulmonary opacities during osimertinib treatment and its clinical implication. J. Thorac. Oncol..

[35] Lamberti G, Andrini E, Ricciuti B (2019). Impact of performance status and age on osimertinib efficacy in patients with *EGFR*-mutant T790M-positive non-small-cell lung cancer. J. Thorac. Dis..

