# Supplementary material for: First-line osimertinib in elderly patients with epidermal growth factor receptor-mutated advanced non-small cell lung cancer: a retrospective multicenter study (HOT2002)
Source: Sci Rep. 2021 Nov 30;11:23140. doi: 10.1038/s41598-021-02561-z (PMC8632978; doi:10.1038/s41598-021-02561-z)
Supplement: Supplementary file 1 — Supplementary Figures. [file 41598_2021_2561_MOESM1_ESM.docx]

**Figure S1.** Kaplan-Meier curves of time to treatment failure (TTF) in all patients

**Figure S2.** Kaplan-Meier curves of progression-free survival (PFS) according to EGFR exon 19 deletion and L858R mutation. Patients with EGFR exon 19 deletion or L858R alone, or co-occurring with other EGFR mutations are included. CI, confidence interval; EGFR, epidermal growth factor receptor; NR, not reached.

**Figure S3.** Kaplan–Meier curves of progression-free survival (PFS) according to common and uncommon mutations in EGFR. Common mutations include EGFR exon 19 deletion or L858R alone, or co-occurring with other EGFR mutations. Uncommon mutations include mutations other than the common mutations (3 cases of G719X, 1 case of L861Q, and 1 case of exon 20 insertion). One patient having a compound mutation with exon 19 deletion and L858R was excluded from the analysis. CI, confidence interval; EGFR, epidermal growth factor receptor; NR, not reached.

**Figure S4.** Best overall response to osimertinib. The maximum percentage change in measurable tumor target lesions (using Response Evaluation Criteria in Solid Tumors [RECIST], version 1.1) at the time of best response from baseline. (A) All patients, (B) patients who had dose reductions within the first 3 months, and (C) those who received osimertinib 80 mg daily for first 3 months.

**Figure S5.** Kaplan­­–Meier curves of progression-free survival (PFS) in patients with or without dose reduction within 3 months, including patients who discontinued within initial 3 months. CI, confidence interval; NR, not reached.

**Figure S6.** Kaplan–Meier curves of time to treatment failure (TTF) in patients with or without pneumonitis. CI, confidence interval; NR, not reached.
