# Supplementary material for: First-line osimertinib in elderly patients with epidermal growth factor receptor-mutated advanced non-small cell lung cancer: a retrospective multicenter study (HOT2002)
Source: Sci Rep. 2021 Nov 30;11:23140. doi: 10.1038/s41598-021-02561-z (PMC8632978; doi:10.1038/s41598-021-02561-z)
Supplement: Supplementary file 2 — Supplementary Tables. [file 41598_2021_2561_MOESM2_ESM.docx]

**Table S1. Comparison of baseline characteristics between PS 0-1 and PS ≥ 2**

| Characteristic | PS 0-1 (n=113) | PS ≥2 (n=19) | p Values |
| --- | --- | --- | --- |
| Median age (range), y | 80 (75-90) | 82 (75-88) | 0.08 |
| Age group, n (%) |  |  |  |
| 75-79 years | 55 (48.7) | 8 (42.1) |  |
| 80-84 years | 41 (36.3) | 3 (15.8) |  |
| ≥ 85 years | 17 (15.0) | 8 (42.1) | 0.014 |
| Sex, n (%) |  |  |  |
| Male | 32 (28.3) | 6 (31.6) |  |
| Female | 81 (71.7) | 13 (68.4) | 0.77 |
| Pathology, n (%) |  |  |  |
| Adenocarcinoma | 113 (100) | 19 (100) |  |
| Stage, n (%) |  |  |  |
| IIIA | 2 (1.8) | 0 |  |
| IIIB | 3 (2.7) | 0 |  |
| IVA | 35 (31.0) | 2 (10.5) |  |
| IVB | 40 (35.4) | 16 (84.2) |  |
| Recurrent | 33 (29.2) | 1 (5.3) | 0.003 |
| ECOG performance status, n (%) | |  |  |
| 0 | 30 | – |  |
| 1 | 83 | – |  |
| 2 | – | 15 |  |
| 3 | – | 3 |  |
| 4 | – | 1 |  |
| CNS metastasis, n (%) |  |  |  |
| Present | 21 (18.6) | 6 (31.6) |  |
| Absent | 92 (81.4) | 13 (68.4) | 0.19 |
| Liver metastasis, n (%) |  |  |  |
| Present | 6 (5.3) | 4 (21.1) |  |
| Absent | 107 (94.7) | 15 (79.0) | 0.016 |
| Smoking history, n (%) |  |  |  |
| Yes | 33 (29.2) | 7 (36.8) |  |
| No | 80 (70.8) | 12 (63.2) | 0.5 |
| EGFR mutation, n (%) |  |  |  |
| exon 19 deletion | 39 (34.5) | 6 (31.6) |  |
| exon 19 deletion + T790M | 0 | 1 (5.3) |  |
| L858R | 66 (58.4) | 9 (47.4) |  |
| L858R + T790M | 2 (1.8) | 2 (10.5) |  |
| L858R + S768I | 0 | 1 (5.3) |  |
| exon 19 deletion + L858R | 1 (0.9) | 0 |  |
| G719X | 3 (2.6) | 0 |  |
| L861Q | 1 (0.9) | 0 |  |
| exon 20 insertion | 1 (0.9) | 0 | 0.79 |
| CCI, n (%) |  |  |  |
| CCI < 2 | 84 (74.3) | 14 (73.7) |  |
| CCI ≥ 2 | 29 (25.7) | 5 (26.3) | 0.95 |
| BSA |  |  |  |
| < 1.5 | 76 (67.3) | 12 (63.2) |  |
| ≥ 1.5 | 36 (31.9) | 6 (31.6) |  |
| NA | 1 (0.9) | 1 (5.26) | 0.35 |

Abbreviations: BSA, body surface area; CCI, Charlson comorbidity index; CNS, central nervous system; ECOG, Eastern Cooperative Oncology Group; EGFR, epidermal growth factor receptor.

**Table S2.** Univariate and multivariate analyses for progression-free survival

| Parameter | Category | Univariable |  | Multivariable |  |
| --- | --- | --- | --- | --- | --- |
|  |  | HR^a^ | *p* value | HR | *p* value |
| Age | ≥ 80 (ref, 75–79) years | 0.68 (0.55–1.46) | 0.66 |  |  |
| Sex | female (ref, male) | 1.04 (0.61–1.78) | 0.89 |  |  |
| Smoking history | no (ref, yes) | 0.87 (0.51–1.48) | 0.61 | 0.74 (0.42–1.30) | 0.300 |
| Clinical stage | recurrence (ref, III or IV) | 0.64 (0.36–1.16) | 0.143 | 0.53 (0.27–1.02) | 0.057 |
| Performance status | ≥ 2 (ref, 0–1) | 1.96 (1.02–3.78) | 0.044 | 1.66 (0.83–3.32) | 0.150 |
| Brain metastasis | absent (ref, present) | 0.80 (0.44–1.45) | 0.46 | 1.04 (0.55–1.95) | 0.910 |
| EGFR mutation | L858R (ref, exon 19 del)^b^ | 1.11 (0.66–1.87) | 0.69 | 1.17 (0.69–1.97) | 0.560 |
|  | Uncommon (ref, common)^c^ | 0.67 (0.16–2.73) | 0.57 |  |  |
| CCI | ≥ 2 (ref, 0–1) | 1.05 (0.59–1.85) | 0.87 | 0.90 (0.49–1.67) | 0.750 |
| BSA | ≥ 1.5 (ref, < 1.5) | 0.84 (0.49–1.42) | 0.50 |  |  |

^a^HRs and 95 % CIs were estimated using the Cox proportional hazards regression model. Without considering the results of the univariate analysis, important factors, according to previous reports and based on medical perspective, were selected for inclusion in the multivariate analysis.

^b^Patients who have an uncommon mutation alone and one patient having a compound mutation with exon 19 deletion and L858R were excluded from the analysis.

^c^Common mutations include EGFR exon 19 deletion and L858R alone, or co-occurring with other EGFR mutations. Uncommon mutations include mutations other than the common mutations. One patient having a compound mutation with exon 19 deletion and L858R was excluded from the analysis.

Abbreviations: BSA, body surface area; CCI, Charlson comorbidity index; CI, confidence interval; EGFR, epidermal growth factor receptor; HR, hazard ratio; ref, reference.

**Table S3.** Baseline characteristics of patients with and without pneumonitis

| Characteristic | Patients with pneumonitis | Patients without pneumonitis | *p* value |
| --- | --- | --- | --- |
| Age, years |  |  | 0.460 |
| Median (range) | 79 (75–89) | 80 (75–90) |  |
| Sex, n (%) |  |  | 0.410 |
| Male | 5 (21.7) | 33 (30.3) |  |
| Female | 18 (78.3) | 76 (69.7) |  |
| Smoking history, n (%) |  |  | 0.610 |
| yes | 8 (34.8) | 32 (29.4) |  |
| no | 15 (65.2) | 77 (70.6) |  |
| Stage, n (%) |  |  | 0.970 |
| III/IV | 17 (73.9) | 81 (74.3) |  |
| Recurrence | 6 (26.1) | 28 (25.7) |  |
| ECOG performance status, n (%) |  |  | 0.131 |
| 0–1 | 22 (95.7) | 91 (83.5) |  |
| ≥ 2 | 1 (4.3) | 18 (16.5) |  |
| CNS metastasis, n (%) |  |  | 0.870 |
| Present | 5 (21.7) | 22 (20.2) |  |
| Absent | 18 (78.3) | 87 (79.8) |  |
| EGFR mutation^a^, n (%) |  |  | 0.770 |
| Exon 19 deletion | 9 (39.1) | 37 (35.9) |  |
| L858R | 14 (60.9) | 66 (64.1) |  |
| CCI, n (%) |  |  | 0.125 |
| CCI < 2 | 20 (87.0) | 78 (71.6) |  |
| CCI ≥ 2 | 3 (13.0) | 31 (28.4) |  |
| BSA^b^ (Du Bois Method), n (%) |  |  | 0.830 |
| BSA < 1.5 | 16 (69.6) | 72 (67.3) |  |
| BSA ≥ 1.5 | 7 (30.4) | 35 (32.7) |  |

^a^Five patients with uncommon mutations and one patient with both exon 19 deletion and L858R mutation were excluded from this subgroup analysis.

^b^Patients whose data on height or weight were not available were excluded from this subgroup analysis.

Abbreviations: BSA, body surface area; CCI, Charlson comorbidity index; CNS, central nervous system; ECOG, Eastern Cooperative Oncology Group; EGFR, epidermal growth factor receptor.

**Table S4.** The number of patients with dose reductions

| **Dosing** | **n (%)** |
| --- | --- |
| No reduction | 78 (59.1) |
| Dose reduction | 54 (40.9) |
| Once | 42 (31.8) |
| 2 times | 12 (9.1) |
| Dose reduction within 3 months | 32 (24) |

**Table S5.** Reasons for treatment discontinuation

|  | **n (%)** |
| --- | --- |
| **Treatment discontinuation** | 70 (53.0) |
| **Reason for discontinuation** |  |
| Progressive disease | 35 (26.5) |
| Adverse events | 35 (26.5) |
| Pneumonitis (All) | 20 (15.2) |
| Pneumonitis (Grade 5) | 2 (1.5) |
| Pneumonitis (Grade 4) | 1 (0.8) |
| Pneumonitis (Grade 3) | 9 (6.8)^a^ |
| Pneumonitis (Grade 2) | 6 (4.5) |
| Pneumonitis (Grade 1) | 2 (1.5) |
| Nausea (All) | 2 (1.5) |
| Nausea (Grade 3) | 1 (0.8) |
| Nausea (Grade 2) | 1 (0.8) |
| Anorexia (All) | 4 (3.0) |
| Anorexia (Grade 3) | 3(2.3) |
| Anorexia (Grade 2) | 1(0.8) |
| Fatigue (All) | 2 (1.5) |
| Fatigue (Grade 3) | 1 (0.8) |
| Fatigue (Grade 2) | 1 (0.8) |
| Diarrhea (Grade 1) | 1 (0.8) |
| Dysgeusia (Grade 2) | 2 (1.5) |
| Rash (Grade 2) | 1 (0.8) |
| Creatinine increased (Grade 2) | 1 (0.8) |
| Heart failure (Grade 5) | 1 (0.8) |
| Cardiac disorders-other; Takotsubo cardiomyopathy (Grade 2) | 1 (0.8) |
| Aortic valve disease (Grade 5) | 1 (0.8) |

^a^One patient had grade 3 pneumonitis at the time of discontinuation, but eventually she died of pneumonitis 6 months after the discontinuation of treatment (grade 5).

**Table S6.** Causes of death

|  | **n (%)** |
| --- | --- |
| **Death (all cause)** | 38 (28.8) |
| **Death due to cancer progression** | 31 (23) |
| **Treatment-related death** | 4 (3) |
| Pneumonitis | 3 (2) |
| Heart failure | 1 (1) |
| **Death due to other cause** | 3 (2) |
| Aortic valve disease | 1 (1) |
| Pulmonary thromboembolism | 1 (1) |
| Heart failure | 1 (1) |

**Table S7.** A summary of reports regarding EGFR-TKI treatments in elderly patients with TKI naïve EGFR-mutated non-small cell lung cancer

| Author | EGFR-TKI | Study  design | Age,  years | Median (range)  age, years | PFS,  months | OS,  months | ORR,  % | Pneumonitis,  Any(≥ G3), % |
| --- | --- | --- | --- | --- | --- | --- | --- | --- |
| Maemondo et al.^13^ (n = 31) | GEF | Pro | ≥ 75 | 80.3(75–89) | 12.3 | 33.8 | 74.0 | 3.2(3.2) |
| Fujita et al.^14^ (n = 22) | GEF | Pro | ≥ 70 | 81(71–85) | 9.7 | 27.9 | 45.5 | 0(0) |
| Takahashi et al.^15^ (n = 20) | GEF | Pro | ≥ 70 | 79.5(72–90) | 10 | 26.4 | 70.0 | 5(0) |
| Uruga et al.^16^ (n = 9) | GEF | Retro | ≥ 70 | 79(73–89) | 13.1 | 17.2 | 66.7 | 0(0) |
| Tateishi et al.^17^ (n = 55) | GEF | Retro | ≥ 75 | 81.1(75–94) | 13.8 | 29.1 | 72.7 | 5.5(0) |
| Kuwako et al.^18^ (n = 62) | GEF | Retro | ≥ 75 | 80(75–89) | 13.2 | 19 | 61.3 | 4.8(1.8) |
| Morikawa et al.^19^ (n = 71) | GEF | Retro^a^ | ≥ 70 | 75(70–89) | 14.3 | 30.8 | 73.2 | NA(2.8) |
| Inoue et al.^20^ (n = 32) | ERL | Pro^b^ | ≥ 75 | 80(75–87) | 15.5 | NR | 56.3 | 9.4(3.1) |
| Miyamoto et al.^21^ (n = 80) | ERL | Pro | ≥ 75^C^ | 80(49–70) | 9.3 | 26.2 | 60.0 | 0(0) |
| Wu et al.^22^ (n = 19) | AFA | Pro | ≥ 75 | 79(75–86) | 14.7 | 27.9 | NA | NA |
| Imai et al.^23^ (n = 40) | AFA | Pro | ≥ 70 | 77(70–85) | 12.9 | NR | 72.5 | 10(7.5) |
| Minegishi et al.^24^ (n = 38) | AFA | Pro | ≥ 75 | 77.5(75–91) | 14.2 | 35.2 | 75.7 | 13.2(5.3) |
| This study (n = 132) | OSI | Retro | ≥ 75 | 80(75–90) | 19.4 | NR | 75.2 | 17.4(9.1) |

^a^Pooled analysis, ^b^subgroup analysis, ^c^including patients aged 20 to 74 years with an age-adjusted CCI of 6 points or higher and/or PS of 2 or greater.

Abbreviations: CCI, Charlson comorbidity index; EGFR, epidermal growth factor receptor; NR, not reached; NA, not available; GEF, gefitinib; ERL, erlotinib; AFA, afatinib; OSI, osimertinib; ORR, objective response rate; OS, overall survival; PFS, progression-free survival; Pro, prospective; Retro, retrospective.
